# The Association between Antenatal Tea Drinking and Hypertensive Disorders of Pregnancy: A Systematic Review and Meta-Analysis

**DOI:** 10.3390/epidemiologia5020014

**Published:** 2024-04-30

**Authors:** Ahmed Arafa, Haytham A. Sheerah, Omar Khaled Alzaydan, Yasser Sabr

**Affiliations:** 1 Department of Preventive Cardiology, National Cerebral and Cardiovascular Center, Suita 564-8565, Japan; 2 Department of Public Health, Faculty of Medicine, Beni-Suef University, Beni-Suef 2721562, Egypt; 3 Office of the Vice Minister of Health, Ministry of Health, Riyadh 12631, Saudi Arabia; hasheerah@moh.gov.sa; 4 King Saud University Medical City, King Saud University, Riyadh 12372, Saudi Arabia; oalzaydan@ksu.edu.sa (O.K.A.); ysabr@ksu.edu.sa (Y.S.); 5 Department of Obstetrics and Gynecology, College of Medicine, King Saud University, Riyadh 11472, Saudi Arabia

**Keywords:** tea, hypertensive disorders of pregnancy, pre-eclampsia, meta-analysis

## Abstract

Several adverse maternal outcomes have been linked to the excessive consumption of caffeine during pregnancy. Tea is an important source of caffeine. Hypertensive disorders of pregnancy (HDP) are common pregnancy complications with unfavorable maternal and fetal complications. This study aimed to investigate the relationship between antenatal tea drinking and HDP using a meta-analysis of available evidence. We systematically retrieved eligible studies before computing the pooled odds ratio (OR) and 95% confidence interval (CI) of HDP for women who reported the highest versus the lowest frequencies of antenatal tea drinking. We used the *I*^2^ statistic to measure heterogeneity across studies and the test for funnel plot asymmetry to evaluate publication bias. The results showed that the highest frequencies of antenatal tea drinking were associated with increased odds of HDP (pooled OR = 1.16, 95% CI: 1.01, 1.33). We identified no signs of heterogeneity across studies (*I*^2^ = 0.0% and p-heterogeneity = 0.498) or publication bias (z = 0.791 and p-publication bias = 0.429). When the outcome was limited to (pre-)eclampsia, the association became statistically non-significant (pooled OR = 1.28, 95% CI: 0.86, 1.89, *I*^2^ = 0.0%, and p-heterogeneity = 0.751). In conclusion, our results indicated that the highest frequency of antenatal tea drinking was positively associated with HDP. Pregnant women should be advised against excessive tea consumption. Still, future prospective cohort studies, considering the effects of different tea types and caffeine content, are needed to confirm our conclusions.

## 1. Introduction

Hypertensive disorders of pregnancy (HDP) stand out as one of the most considerable health worries during pregnancy [1,2]. HDP refers to an umbrella term involving chronic hypertension, gestational hypertension, (pre-)eclampsia, and chronic hypertension with superimposed pre-eclampsia [1,2]. Chronic hypertension occurs when a woman has high blood pressure even before becoming pregnant. Gestational hypertension, on the other hand, develops during pregnancy without any signs of proteinuria or other organ complications. (Pre-)eclampsia is a more severe form of HDP, characterized by high blood pressure and signs of damage to other organs, often accompanied by proteinuria. Chronic hypertension with superimposed pre-eclampsia involves pregnant women with pre-existing high blood pressure who then develop the signs of (pre-)eclampsia [1,2]. These conditions have been linked to unfavorable outcomes for both mothers and infants, as well as potential long-term complications in the offspring [3]. The risks associated with HDP include intrauterine growth restriction, low birth weight, stillbirth, neonatal death, and prolonged maternal hospitalization [4,5]. Moreover, women experiencing HDP face an increased likelihood of future chronic hypertension, coronary heart disease, and heart failure [6], while their children may be more susceptible to autism spectrum disorders, attention-deficit hyperactivity disorder, and diabetes [7,8].

On the other hand, tea, one of the most consumed beverages worldwide, has been enjoyed for centuries for its taste, aroma, and various health benefits. Tea contains various bioactive compounds, including caffeine, catechins, and polyphenols, which have attracted considerable attention for their potential health effects [9]. Caffeine, in particular, is a central nervous system stimulant that can also cross the placenta during pregnancy. It can increase heart rate and blood pressure, and concerns have been raised about its potential impact on maternal and fetal health [10]. Excessive caffeine intake during pregnancy was shown to be associated with miscarriage, stillbirth, low birth weight, and childhood obesity [11,12].

However, the potential association between tea consumption during pregnancy and HDP remains a subject of ongoing debate. A handful of studies have explored this relationship, but they were limited by the small number of HDP cases, and their findings were inconsistent [13,14,15,16,17]. Herein, we aimed to investigate the association between tea drinking during pregnancy and HDP using a systematic review and meta-analysis of the available evidence.

## 2. Methods

The study protocol was not registered with the International Prospective Register of Systematic Reviews (PROSPERO) or any other online database.

### 2.1. Literature Search

In accordance with the guidelines outlined in the Preferred Reporting Items for Systematic Reviews and Meta-Analysis (PRISMA) checklist [18], we conducted this meta-analysis. Two investigators independently conducted comprehensive searches of the Medline (PubMed), Scopus, and Web of Science databases to identify relevant studies published prior to 30 August 2023 (Supplementary Table S1). There were no restrictions placed on the publication year. Additionally, while no specific endeavors were made to obtain unpublished data, a manual examination of the reference lists of retrieved studies and related review articles was undertaken to uncover any potential additional studies.

### 2.2. Eligibility Criteria

Studies were selected for this meta-analysis if they met the following criteria: (1) the exposure was drinking tea during pregnancy, (2) the outcome was HDP, and (3) the risk or prevalence of HDP from drinking tea during pregnancy was shown.

### 2.3. Study Selection

**Figure 1:**
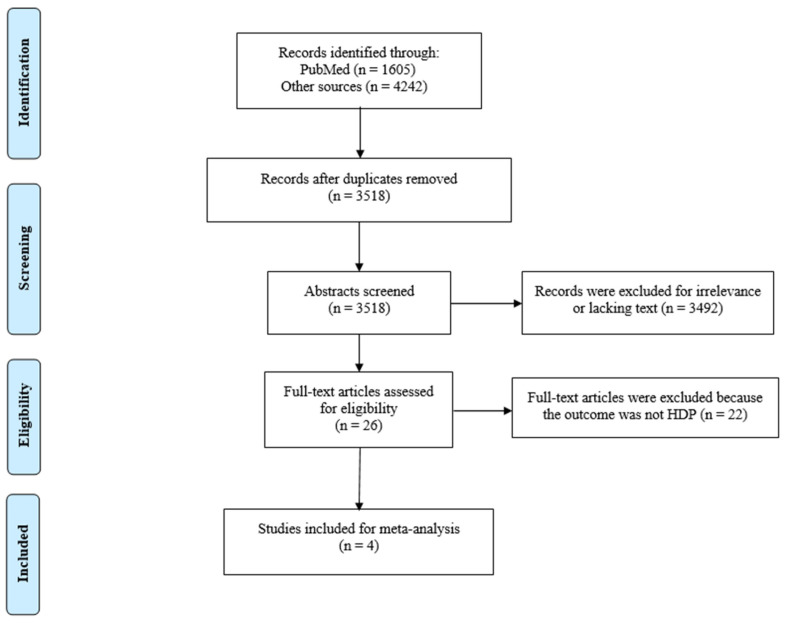
PRISMA flow diagram of the study selection process for the meta-analysis.

We reviewed the titles and abstracts of all studies extracted by the primary search. Then, we subjected these studies to the previously mentioned eligibility criteria to create a final list of studies for meta-analysis. Most studies were excluded because they were either duplicates or reviews or had irrelevant exposures or outcomes (drinking tea during pregnancy was not the exposure and/or HDP or any of its subtypes were not the outcome). Finally, we obtained four studies that were eligible for this meta-analysis (Figure 1). The study selection was conducted by two investigators, and conflicts were solved via discussion with all investigators.

### 2.4. Data Extraction

Two investigators independently extracted the following data from the included studies: the last name of the first author, the year of publication, study area, sample size, study design, HDP definition, categories of drinking tea, adjusted variables, exclusion criteria, and odds ratios (ORs) with corresponding 95% confidence intervals (CIs). When a given study provided different regression models that adjusted for several variables, we included the model that adjusted for the largest number of variables to reduce the risk of confounding bias.

### 2.5. Risk of Bias and Quality Assessment

The quality of the individual studies was determined using the modified Newcastle–Ottawa Scale [19]. Two investigators independently assessed the quality of the studies, with disagreement solved via discussion and consulting the remaining authors.

### 2.6. Statistical Analysis

The ORs and their CIs were used as measures of the association. The random effects model was applied to compute the pooled OR of HDP among women who reported the highest versus the lowest frequencies of drinking tea during pregnancy [20]. We calculated the *I*^2^ to examine statistical heterogeneity across studies [21]. Possible publication bias was assessed via the regression test for funnel plot asymmetry [22]. We used the R 3.2.0 statistical package (Metafor: Meta-Analysis Package for R) to conduct the meta-analysis [23].

## 3. Results

This meta-analysis included four observational studies investigating women from Canada, Iran, Japan, and China. The studies were published between 2009 and 2022. The association of drinking tea during pregnancy with pregnancy-induced hypertension (PIH) was calculated in two studies [16,17], and the association with (pre-)eclampsia was calculated in three studies [13,14,17] (Table 1).

Wei and colleagues investigated nulliparous pregnant women in Quebec, Canada (92 women with pre-eclampsia and 245 normotensive controls) between January 2003 and March 2006. Participants, both cases and controls, were recruited for the study within 48 h following delivery. Eligibility criteria for enrollment included women aged at least 18 years, nulliparous, and proficient in either French or English. Exclusion criteria encompassed multiparous women, those with chronic hypertension or hypertension prior to 20 weeks of pregnancy, gestational hypertension without proteinuria, pregestational diabetes, heart disorders, or positive HIV serology. Compared with non-tea drinking during pregnancy, the ORs (95% CIs) of pre-eclampsia for tea drinking were 1.39 (95% CI, 0.81, 2.41) for any drinking, 1.37 (95% CI, 0.67, 1.81) for tea drinking <20 weeks only, 1.18 (0.34, 4.05) for tea drinking ≥20 weeks only, and 1.88 (1.01, 3.51) for persistent tea drinking. The corresponding results of the dose–response analyses were 1.36 (0.71, 2.60) for <3 cups/day, 1.43 (0.50, 4.13) for 3–7 cups/day, and 1.81 (0.53, 6.12) for ≥7 cups/day [13]. Still, the study had some limitations that should be mentioned. For example, the study did not gather information on the specific type of tea consumed, the concentrations of catechin and caffeine present in the tea, or whether it was consumed with milk or other additives. Additionally, data on tea intake relied on maternal self-reporting, potentially introducing recall bias.

Sharbaf and colleagues compared 40 women with pre-eclampsia to 100 healthy pregnant women at the Women’s Medical Center and Valiasr General Hospital in Tehran, Iran from November 2009 to December 2010. Cases and controls were recruited to the study within 48 h after delivery. Women had to be at least 18 years old to be included. Multiparous women and those with chronic hypertension, heart disorder, HIV-positive serology, or a history of intrauterine fetal death or abortion were excluded. Their study showed no differences between groups regarding antenatal tea drinking. Among the 40 women with pre-eclampsia, 35 women (87.5%) reported drinking ≤3 cups of tea/day and 5 women (12.5%) reported drinking >3 cups of tea/day. Among the 100 women who served as controls, 87 women (87.0%) reported drinking ≤3 cups of tea/day and 13 women (13.0%) reported drinking >3 cups of tea/day [14]. In addition to the high likelihood of recall bias, this study had several limitations, such as the very limited sample size, not adjusting results for several confounders, and using a very high threshold to categorize the consumption of tea during pregnancy.

Kawanishi et al. investigated 85,533 singleton pregnant women with live births from the Japan Environment and Children’s Study, a prospective cohort involving women from early pregnancy onward. Women without subsequent delivery records and cases of multiple pregnancies, stillbirth, abortion, diabetes mellitus, gestational diabetes mellitus, hypertension, renal disease, or a history of HDP were excluded. Compared to non-tea drinking, the ORs (95% CIs) for HDP were 1.04 (0.92, 1.17) for <1 cup/day, 1.19 (1.03, 1.36) for 1 cup/day, and 1.11 (0.95, 1.29) for ≥2 cups/day. In terms of limitations, this study lacked comprehensive details on categories of HDP, such as gestational hypertension and pre-eclampsia. Furthermore, the study did not gather data on the frequency of consumption of decaffeinated green tea and oolong tea, which are commonly consumed in Japan [16]. Li et al. performed a retrospective birth cohort study using data from 10,452 women collected between 2010 and 2012 at the Gansu Provincial Maternity and Child Care Hospital in Lanzhou, China. Participants eligible for inclusion were those who had visited the hospital for delivery at a gestational age of 20 weeks or more. All included participants were free from mental illness and were aged 18 years or older. Women who were multiparous, had experienced stillbirth or abortion, or had a medical history of hypertension, renal disease, or pelvic inflammatory disease in previous pregnancies were excluded from the study. The study concluded that tea consumption during pregnancy was significantly associated with an increased risk of PIH, gestational hypertension, and early onset pre-eclampsia: ORs (95% CIs) = 1.44 (1.01, 2.05), 1.86, (1.07, 3.21), and 2.93 (1.21, 7.09), respectively [17]. However, the study had some limitations. For instance, data regarding tea consumption were gathered through in-person interviews conducted within two days before delivery or three days after delivery, potentially introducing recall bias. Furthermore, the authors did not acquire precise information regarding the specific quantities of tea consumed by each individual.

**Figure 2:**
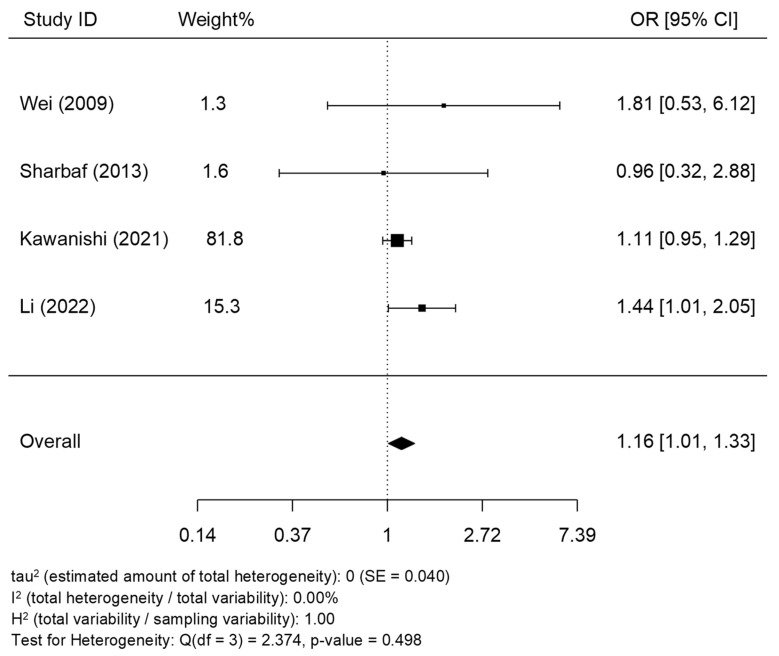
Meta-analysis of the association between drinking tea during pregnancy and hypertensive disorders of pregnancy [13,14,16,17].

**Figure 3:**
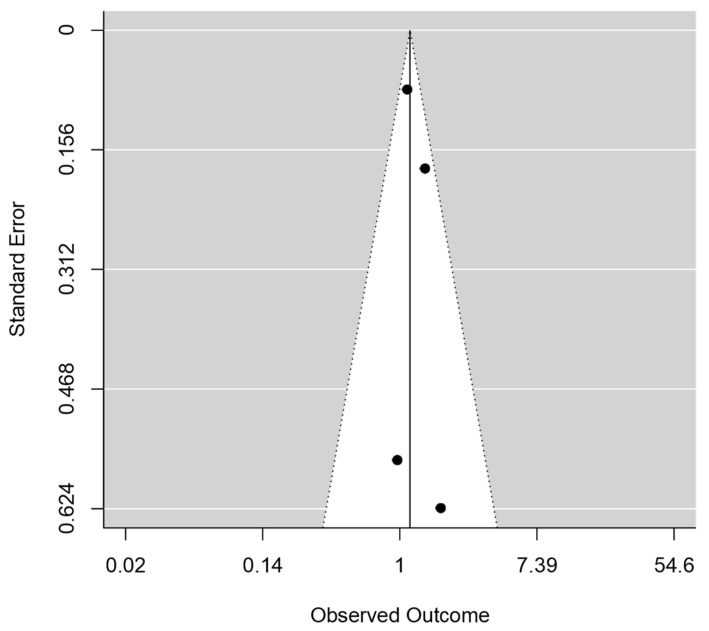
Funnel plot showing publication bias in studies that assessed the association between antenatal tea consumption and hypertensive disorders of pregnancy.

**Figure 4:**
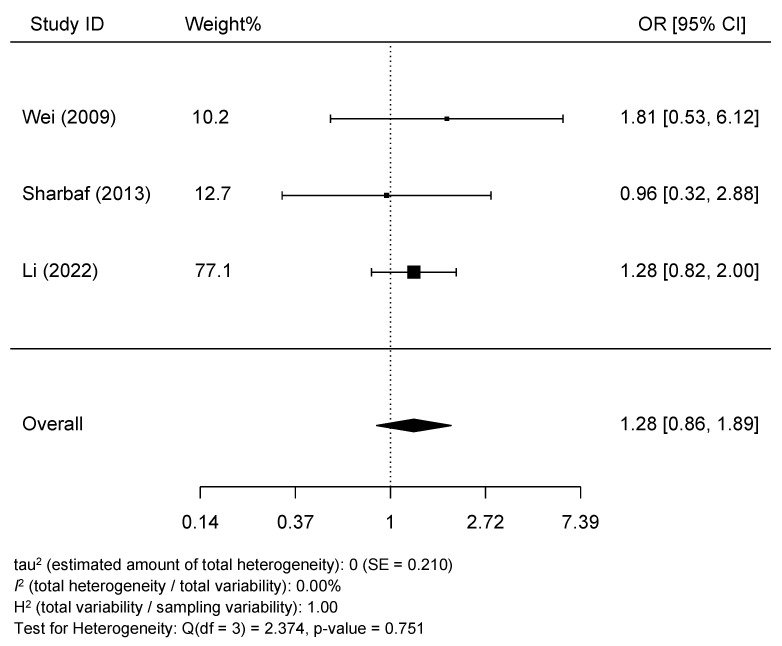
Meta-analysis of the association between drinking tea during pregnancy and pre(-eclampsia) [13,14,17].

Only one study showed statistically significant increased odds of HDP with tea drinking during pregnancy [17], while the association was not statistically significant in three studies [13,14,16]. Together, women who reported the highest frequency of drinking tea during pregnancy showed increased odds of HDP (pooled OR = 1.16, 95% CI: 1.01, 1.33). We detected no signs of heterogeneity across studies (*I*^2^ = 0.0% and p-heterogeneity = 0.498) (Figure 2). Besides, no publication bias was shown (z = 0.791 and p-publication bias = 0.429) (Figure 3). In addition, the association became statistically non-significant with (pre-)eclampsia (n = 3, pooled OR = 1.28, 95% CI: 0.86, 1.89, *I*^2^ = 0.0%, and p-heterogeneity = 0.751) (Figure 4). According to the modified Newcastle–Ottawa Scale, three studies had good quality with a low risk of bias [13,16,17], while one study showed a high likelihood of bias [14] (Table 2). However, the study with the highest bias contributed to only 1.6% of the weight of the meta-analysis and showed no association between tea consumption during pregnancy and HDP, suggesting that it did not affect the conclusion.

## 4. Discussion

Investigating the association between antenatal tea consumption and HDP holds importance because understanding potential risk factors for HDP is essential for preventive healthcare, especially since tea consumption is a modifiable lifestyle behavior. Furthermore, healthcare professionals could be able to provide tailored advice to pregnant women about their tea intake, potentially reducing the incidence of HDP. Additionally, raising awareness among pregnant women about the potential effects of tea consumption on their health may empower them to make informed choices about their dietary habits during pregnancy.

This meta-analysis revealed that women who reported the highest frequencies of tea consumption during pregnancy were more likely to have HDP, with no evidence of heterogeneity across studies or publication bias. To the best of our knowledge, this is the first meta-analysis to investigate this association.

The mechanism by which maternal tea consumption could result in HDP is obscure. However, caffeine, a major active chemical in tea that passes the placenta freely, could have hypertensive effects via several pathways. First, caffeine primarily exerts its effects by antagonizing adenosine receptors in the central nervous system and peripheral tissues, resulting in increased blood pressure [24,25]. Second, caffeine stimulates the release of catecholamines, such as adrenaline and noradrenaline, from the adrenal glands, leading to increased heart rate, cardiac output, and peripheral vasoconstriction [26]. Third, caffeine intake has been shown to stimulate renin release from the kidneys. Renin initiates the conversion of angiotensinogen to angiotensin I, which is subsequently converted to angiotensin II by angiotensin-converting enzyme (ACE). Angiotensin II is a potent vasoconstrictor and also stimulates the release of aldosterone, leading to sodium and water retention, further contributing to increased blood pressure [27]. Fourth, chronic caffeine consumption has been associated with endothelial dysfunction, characterized by impaired endothelium-dependent vasodilation. Endothelial cells play a crucial role in regulating vascular tone by producing nitric oxide (NO), a potent vasodilator. Caffeine-induced endothelial dysfunction reduces NO bioavailability, leading to impaired vasodilation and increased vascular resistance, ultimately raising blood pressure [28]. Fifth, caffeine might impair insulin sensitivity and glucose metabolism [29]. Insulin resistance and dysregulated glucose metabolism are risk factors for hypertension and cardiovascular disease [30]. Sixth, caffeine interferes with intracellular calcium signaling, which plays a crucial role in smooth muscle contraction and vascular tone regulation. By inhibiting phosphodiesterases and promoting calcium release from intracellular stores, caffeine increases intracellular calcium levels, leading to enhanced smooth muscle contractility and vasoconstriction, thereby elevating blood pressure [31].

In pregnant women, the hypertensive effects of caffeine intake may have additional implications due to physiological changes that occur during pregnancy [10]. Pregnancy is characterized by increased plasma volume, cardiac output, and peripheral vasodilation, which may alter the body’s response to caffeine [10]. A study of 7890 pregnant women from the Generation R Study showed that higher caffeine intake during pregnancy was associated with elevated systolic blood pressure [32]. In the Kawanishi et al. study, the OR (95% CI) depicting the association between drinking tea during pregnancy and HDP was attenuated after adjusting for total caffeine intake from 1.24 (1.09, 1.41) to 1.11 (0.95, 1.29), suggesting that caffeine intake partly explained the excess risk [16]. The same study showed that the association with black tea was stronger than that with oolong tea and green tea; ORs (95% CIs) for drinking ≥2 cups of tea/day were 1.33 (0.94, 1.88) for black tea, 1.21 (0.97, 1.51) for oolong tea, and 1.05 (0.90, 1.23) for green tea [16]. Similarly, Li et al. showed a stronger association between black tea and PIH than with green tea and herbal tea; ORs (95% CIs) of PIH for drinking any amount of tea during pregnancy were 3.57 (1.67, 7.62) for black tea, 1.86 (0.94, 3.67) for herbal tea, and 1.50 (0.95, 2.36) for green tea [17]. The caffeine content of black tea is higher than that of oolong and green tea [33]. Still, a recent meta-analysis of ten studies involving 114,984 pregnant women indicated no association between caffeine exposure during pregnancy and the risk of gestational hypertension or pre-eclampsia [34].

On the other hand, the effect of tea on hypertension in non-pregnant populations remains controversial. A meta-analysis of 25 trials, including 1476 participants, showed that acute tea intake had no effects on blood pressure, while long-term tea intake led to reductions in mean systolic and diastolic blood pressure by −1.8 (95% CI: −2.4, −1.1) mmHg and −1.4 (95% CI: −2.2, −0.6) mmHg, respectively [35]. Another meta-analysis of five trials including 408 participants with elevated blood pressure or hypertension indicated reductions in mean systolic and diastolic blood pressure attributed to regular tea intake by −4.81 (95% CI: −8.40, −1.58) mmHg and −1.98 (95% CI: −3.77, −0.20) mmHg, respectively [36]. On the other hand, using data from the Shanghai Women’s Health Study (n = 31,351) and the Shanghai Men’s Health Study (n = 28,342), a cohort study showed a 7% higher risk of hypertension among current tea drinkers and a significant dose–response association between the amount of tea consumed and blood pressure [37].

This meta-analysis has several limitations that should be considered. First, only four studies were included [16]; therefore, more studies are needed to confirm our findings and provide enough statistical power to assess publication bias. Second, the definition of HDP varied across studies. In the Li et al. study, the association between drinking tea during pregnancy and PIH was more robust than that with (pre-)eclampsia: ORs (95% CIs) = 1.44 (1.01, 2.05) with PIH versus 1.28 (0.82, 2.00) with (pre-)eclampsia [17]. Another study including Dutch women showed that drinking an extra cup of tea/day was associated with an increased risk of PIH but not (pre-)eclampsia: ORs (95% CIs) = 1.13 (1.04, 1.23) and 1.04 (0.83, 1.30), respectively [15]. Because of the overlapping definitions of HDP across studies and the limited number of these studies, we were not able to stratify HDP by subtypes. Third, the included studies used different cut-offs for drinking tea to calculate the risk of HDP, which led to an overlapping of tea categories across studies. Thus, we could not determine the safe amount of tea to be consumed during pregnancy. Fourth, it seems that HDP risk could differ based on the duration of drinking tea during pregnancy. Li et al. concluded that drinking tea during the first and second trimesters posed a higher risk of HDP than drinking tea during the third trimester only [17]. Fifth, two studies collected data about the type of tea and showed that the association with HDP was stronger with black tea than with green tea [16,17]. Future studies should stratify their results by tea type. Sixth, since most studies used a retrospective design, we cannot exclude the possibility of recall bias. Seventh, because of the observational nature of the included studies, the presence of undetected confounders is likely. Specifically, Sharbaf et al. did not adjust their results for any confounders, elevating the possibility of bias [14]. Eighth, confining the bibliographic research to three databases and not considering unpublished studies are limitations.

## 5. Conclusions

Our meta-analysis indicated that the highest frequency of tea drinking during pregnancy was associated with increased HDP. Therefore, it is prudent for pregnant women to minimize tea consumption. Still, well-designed prospective cohort studies are needed to establish causality and explore potential dose–response relationships. Studies investigating the effects of specific tea types, brewing methods, and caffeine content on maternal health outcomes are necessary to provide more tailored recommendations for pregnant women. Additionally, more studies to elucidate potential biological explanations of this association are also warranted.

## Figures and Tables

**Table 1:** Summary of the studies included in the meta-analysis.

Study ID	Study Design	HDP Definition	Drinking Categories	Covariates	Exclusions
Wei et al. [13] (2009) Canada	Case–control Cases: 92 Controls: 245	Pre-eclampsia	Never (Ref), 0–3, 3–7, and ≥7 cups/week during the first 20 weeks	Maternal age, body mass index, education, smoking, and history of abortion	<18 years, multiparous, chronic hypertension, hypertension before 20 weeks of pregnancy, gestational hypertension without proteinuria, pregestational diabetes, heart disorders, and HIV-positive serology
Sharbaf et al. [14] (2013) Iran	Case–control Cases: 40 Controls: 100	Pre-eclampsia	≤3 (Ref) and >3 cups/day during the first trimester	NA	<18 or >35 years, multiparous, chronic hypertension, heart disorder, HIV positive serology, and history of intrauterine fetal death or abortion
Kawanishi et al. [16] (2021) Japan	Population-based birth cohort n = 85,533	Pregnancy-induced hypertension	None (Ref), <1, 1 to <2, and ≥2 cups/day during pregnancy	Age, parity, coffee and caffeine intake during pregnancy, pre-pregnancy body mass index, smoking, alcohol consumption, folic acid supplementation, and education	Multiple pregnancies, cases of stillbirth or abortion, cases with a medical history of hypertension, renal disease, history of hypertension in previous pregnancies, and cases of diabetes and gestational diabetes
Li et al. [17] (2022) China	Retrospective cohort n = 10,452	Pregnancy-induced hypertension, gestational hypertension, and pre(-eclampsia)	≤3 (Ref) and >3 cups/week during pregnancy	Maternal age, education, monthly family income, parity, smoking, and gestational weight gain	Multiparous, cases of stillbirth or abortion, having a medical history of hypertension, renal disease, and a history of pelvic inflammatory disease in previous pregnancies

HELLP: hemolysis, elevated liver enzymes, and low platelets.

**Table 2:** Risk of bias assessment using the Newcastle–Ottawa Quality Assessment Scale.

Item	Wei et al.	Sharbaf et al.	Kawanishi et al.	Li et al.
Case definition	*	-	*	*
Representativeness of cases	*	*	*	*
Selection of controls	*	*	*	*
Definition of controls	*	*	*	*
Comparability	**	*	**	**
Ascertainment of exposure	*	*	*	*
The same method of ascertainment for cases and controls	*	*	*	*
Nonresponse rate	-	-	-	-
Overall (total number of asterisks)	8	6	8	8

The possible overall scores (asterisks) range between 0 and 9.

## Data Availability

All data generated or analyzed during this study are included in this published article.

## Supplementary Materials

The following supporting information can be downloaded at https://www.mdpi.com/article/10.3390/epidemiologia5020014/s1. Table S1: PubMed search strategy.
