# Supplementary material for: The Association between Antenatal Tea Drinking and Hypertensive Disorders of Pregnancy: A Systematic Review and Meta-Analysis
Source: Epidemiologia (Basel). 2024 Apr 30;5(2):200–10. doi: 10.3390/epidemiologia5020014 (PMC11130964; doi:10.3390/epidemiologia5020014)
Supplement: Supplementary file 1 [file epidemiologia-05-00014-s001.zip › epidemiologia-2940408-supplementary.pdf]

Supplementary Table 1: PubMed search strategy

Search: (Tea) AND ((Preeclampsia) OR (Pregnancy) OR (Hypertension))<br>("tea"[MeSH Terms] OR "tea"[All Fields]) AND ("pre eclampsia"[MeSH Terms] OR "pre eclampsia"[All Fields] OR "preeclampsia"[All Fields] OR ("pregnancy"[MeSH Terms] OR "pregnancy"[All Fields] OR "pregnancies"[All Fields] OR "pregnancy s"[All Fields]) OR ("hypertense"[All Fields] OR "hypertension"[MeSH Terms] OR "hypertension"[All Fields] OR "hypertension s"[All Fields] OR "hypertensions"[All Fields] OR "hypertensive"[All Fields] OR "hypertensive s"[All Fields] OR "hypertensives"[All Fields]))
Translations<br>Tea: "tea"[MeSH Terms] OR "tea"[All Fields]<br>Preeclampsia: "pre-eclampsia"[MeSH Terms] OR "pre-eclampsia"[All Fields] OR "preeclampsia"[All Fields]<br>Pregnancy: "pregnancy"[MeSH Terms] OR "pregnancy"[All Fields] OR "pregnancies"[All Fields] OR "pregnancy's"[All Fields]<br>Hypertension: "hypertense"[All Fields] OR "hypertension"[MeSH Terms] OR "hypertension"[All Fields] OR "hypertension's"[All Fields] OR "hypertensions"[All Fields] OR "hypertensive"[All Fields] OR "hypertensive's"[All Fields] OR "hypertensives"[All Fields]
